# Description of a new tick species, *Ixodes collaris* n. sp. (Acari: Ixodidae), from bats (Chiroptera: Hipposideridae, Rhinolophidae) in Vietnam

**DOI:** 10.1186/s13071-016-1608-0

**Published:** 2016-06-10

**Authors:** Sándor Hornok, Tamás Görföl, Péter Estók, Vuong Tan Tu, Jenő Kontschán

**Affiliations:** Department of Parasitology and Zoology, Faculty of Veterinary Science, Szent István University, Budapest, Hungary; Department of Zoology, Hungarian Natural History Museum, Budapest, Hungary; Department of Zoology, Eszterházy Károly College, Eger, Hungary; Institute of Ecology and Biological Resources, Vietnam Academy of Science and Technology, Hanoi, Vietnam; Plant Protection Institute, Centre for Agricultural Research, Hungarian Academy of Sciences, Budapest, Hungary

**Keywords:** *Ixodes vespertilionis*, Bat tick, *Rhinolophus affinis*, New species, *Ixodes collaris* Hornok n. sp, Vietnam

## Abstract

**Background:**

In a recent study on ixodid bat ticks from Eurasia, a high genetic difference was found between *Ixodes vespertilionis* from Europe and Vietnam. Accordingly, it was proposed that *I. vespertilionis* is a species complex, with at least one additional, hitherto undescribed species. The aim of the present study was to investigate the morphology of bat ticks from Vietnam and to assess their taxonomic status in comparison with those collected in Europe.

**Findings:**

Ixodid bat ticks (two females and two nymphs) collected from the pomona leaf-nosed bat (*Hipposideros pomona*) (Hipposideridae) and intermediate horseshoe bat (*Rhinolophus affinis*) (Rhinolophidae) in Vietnam showed major morphological differences from European isolates of *I. vespertilionis*, including the shape of the scutum, the enclosure and shape of porose areas, the presence of a caudo-lateral collar-like ridge ventrally on the basis capituli, polytrich coxae with short setae, and grouped (non-linear) arrangement of anterior pit sensillae in Haller’s organ.

**Conclusions:**

In this study the female and the nymph of an ixodid bat tick species from Vietnam are described for the first time. The genetic and morphological differences between *I. vespertilionis* Koch, 1844 and these bat ticks from Vietnam justify the status of the latter as a distinct species, *Ixodes collaris* Hornok n. sp.

## Background

In Eurasia, three bat tick species or their genetic variants appear to be geographically widespread: *Ixodes vespertilionis* Koch, 1844, *I. simplex* Neumann, 1906 and *I. ariadnae* Hornok, 2014 [1]. Concerning *I. vespertilionis*, a high genetic difference was found between specimens collected in Europe and another isolate collected in Vietnam. In particular, in the amplified fragment of their cytochrome *c* oxidase subunit I (COI) gene the genotypes from Europe and Vietnam differed by 16 %, greatly exceeding the level of the reported average COI sequence divergence between species (i.e. 6.1 %: [2]). Accordingly, it was proposed that *I. vespertilionis* may be a species complex, with at least one additional, hitherto undescribed species [1]. The aim of the present study was to investigate the morphological differences between ixodid bat ticks collected in Vietnam and Europe, and to verify the existence of a new species in the *I. vespertilionis* species group.

## Methods

Ticks were removed from bats (caught with mist-nets during biodiversity inventory and monitoring projects) and immediately placed into 96 % ethanol. Molecular genetic analysis was performed as previously reported (paratype No. 2 below; see [1]). Pictures were made with a VHX-5000 (Keyence Co., Osaka, Japan) digital microscope.

## Results

**Family Ixodidae Koch, 1844**

**Genus*Ixodes*Latreille, 1795**

***Ixodes collaris*Hornok n. sp.**

***Type-host*:***Hipposideros pomona* Andersen, 1918 (Hipposideridae), pomona leaf-nosed bat.

***Other hosts*:***Rhinolophus affinis* Horsfield, 1823 (Rhinolophidae), intermediate horseshoe bat.

***Type-locality*:** Ngoc Linh Nature Reserve (15.20598N, 107.7937E), Kon Tum Province, Vietnam.

***Other localities*:** Ba Vi National Park (21.08174N, 105.37534E), Hanoi Province, Vietnam; Phia Oac - Phia Den Nature Reserve (22.56327N, 105.87404E), Cao Bang Province, Vietnam; Phu Lac commune - Tuy Phong District (11.225N, 108.6854E), Binh Thuan Province, Vietnam.

***Type-specimens*:** Holotype: female ex *H. pomona*, collected in Vietnam (1,080 m a.s.l., Ngoc Linh Nature Reserve, Kon Tum Province: 15.20598N, 107.7937E) by Vuong Tan Tu (22.ix.2014); deposited in the Soil Zoological Collection of the Hungarian Natural History Museum (accession number HNHM-PED Ixo-00567). Paratype No. 1: female ex *R. affinis*, collected in Vietnam (418 m a.s.l., Ba Vi National Park, Hanoi Province: 21.08174N, 105.37534E) by Vuong Tan Tu (25.vii.2010); deposited in the Department of Parasitology and Zoology, Faculty of Veterinary Science, Szent István University (accession number UNIVET-PAR-HS108). Paratype No. 2: nymph ex *R. affinis*, collected in Vietnam (Phia Oac - Phia Den Nature Reserve, Cao Bang Province: 22.56327N, 105.87404E) by Tamás Görföl, Péter Estók and Vuong Tan Tu (20.x.2014); deposited in the Department of Parasitology and Zoology, Faculty of Veterinary Science, Szent István University (accession number UNIVET-PAR-HS109). Paratype No. 3: nymph ex *R. affinis*, collected in Vietnam (Phu Lac commune - Tuy Phong District, Binh Thuan Province: 11.225N, 108.6854E) by Vuong Tan Tu (7.xii.2015); deposited in the Institute of Ecology and Biological Resources, Vietnam Academy of Science and Technology (accession number IEBR-VN15-057).

***Representative DNA sequences*:** Mitochondrial cytochrome *c* oxidase subunit I (COI) (GenBank acc. no. KR902756); 16S rRNA gene (GenBank acc. no. KR902771). These sequences were generated from tissues of paratype No. 2.

***ZooBank registration*:** To comply with the regulations set out in article 8.5 of the amended 2012 version of the *International Code of Zoological Nomenclature* (ICZN) [3], details of the new species have been submitted to ZooBank. The Life Science Identifier (LSID) of the article is urn:lsid:zoobank.org:pub:69505966-6D7B-4190-AC05-478555A34C5D. The LSID for the new name *Ixodes collaris* is urn:lsid:zoobank.org:act:4F3017E9-93D1-4C0B-ACF3-26CB11AC8928.

***Etymology*:** The name of the new species refers to the ventral, caudolateral collar-like ridge on the basis capituli, which appears to be a unique character.

### Description

***General.*** Medium-sized prostriate tick species. Scutum elongate, reverse pentagonal in shape, with broadly convex (almost rounded) caudo-lateral edge. Palps moderately elongated, basis capituli dorsally triangular, ventrally with caudo-lateral collar-like ridge. Legs long. Coxae with multiple, short setae in addition to long ones.

**Fig. 1:**
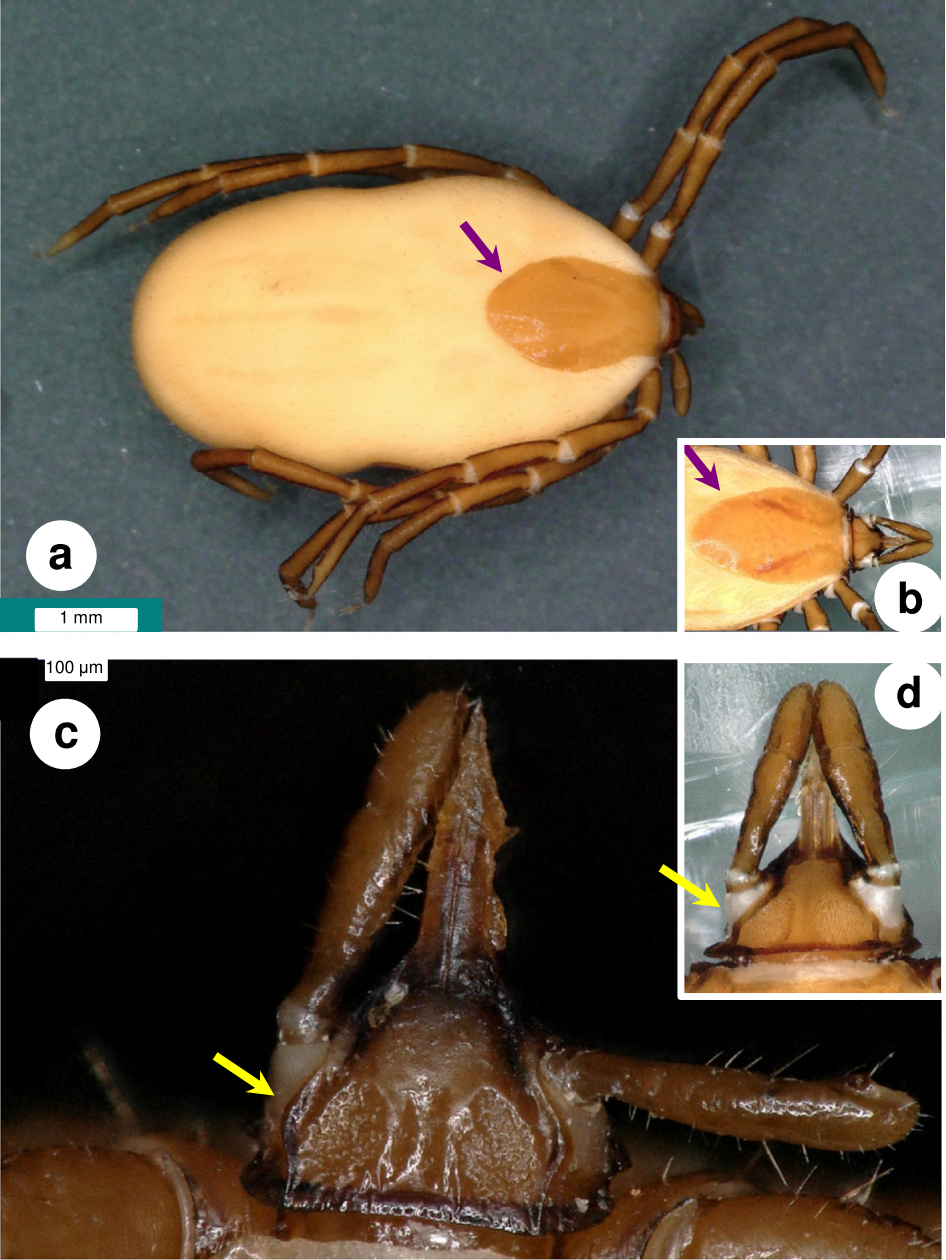
Dorsal view of female of *Ixodes collaris* n. sp. **a** Holotype: posteriorly broad scutum (*arrow*), as contrasted to that of *Ixodes vespertilionis* female (**b**); **c** Basis capituli and palps of paratype No. 1. showing convex longitudinal flanks (*arrow*) enclosing the porose areas, which are longer than broad, as contrasted to those of *I. vespertilionis* female (**d**)

**Fig. 2:**
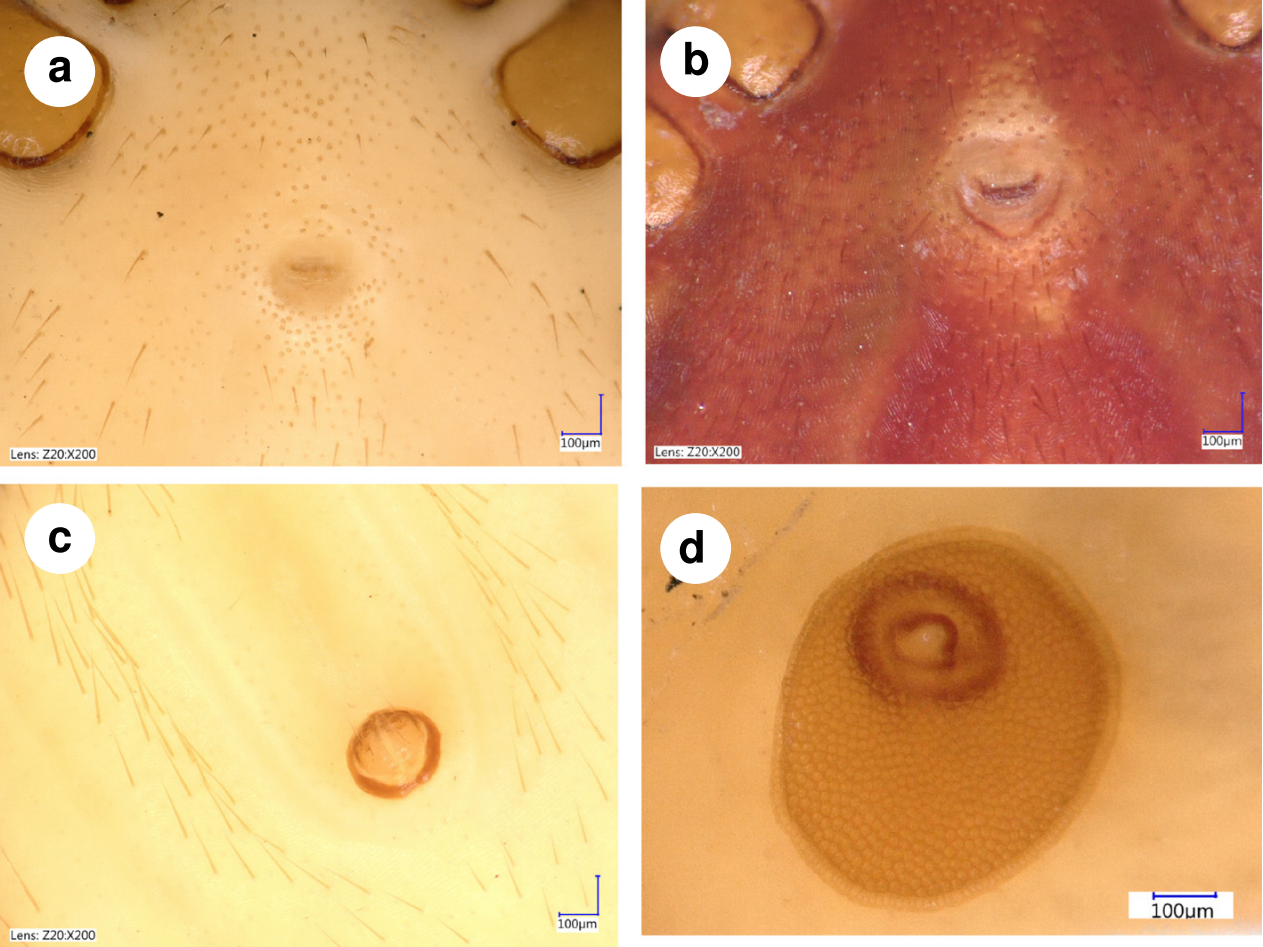
Ventral idiosomal setae of **a** *Ixodes collaris* n. sp. (holotype) and **b** *Ixodes vespertilionis* in a similar state of engorgement. Note that *I. collaris* n. sp. has shorter setae anteriorly to the genital aperture than posteriorly, whereas setae of *I. vespertilionis* are similar in length both anteriorly and posteriorly to the genital aperture. *I. collaris* n. sp.: **c** perianal setae; **d** spiracular plate

**Fig. 3:**
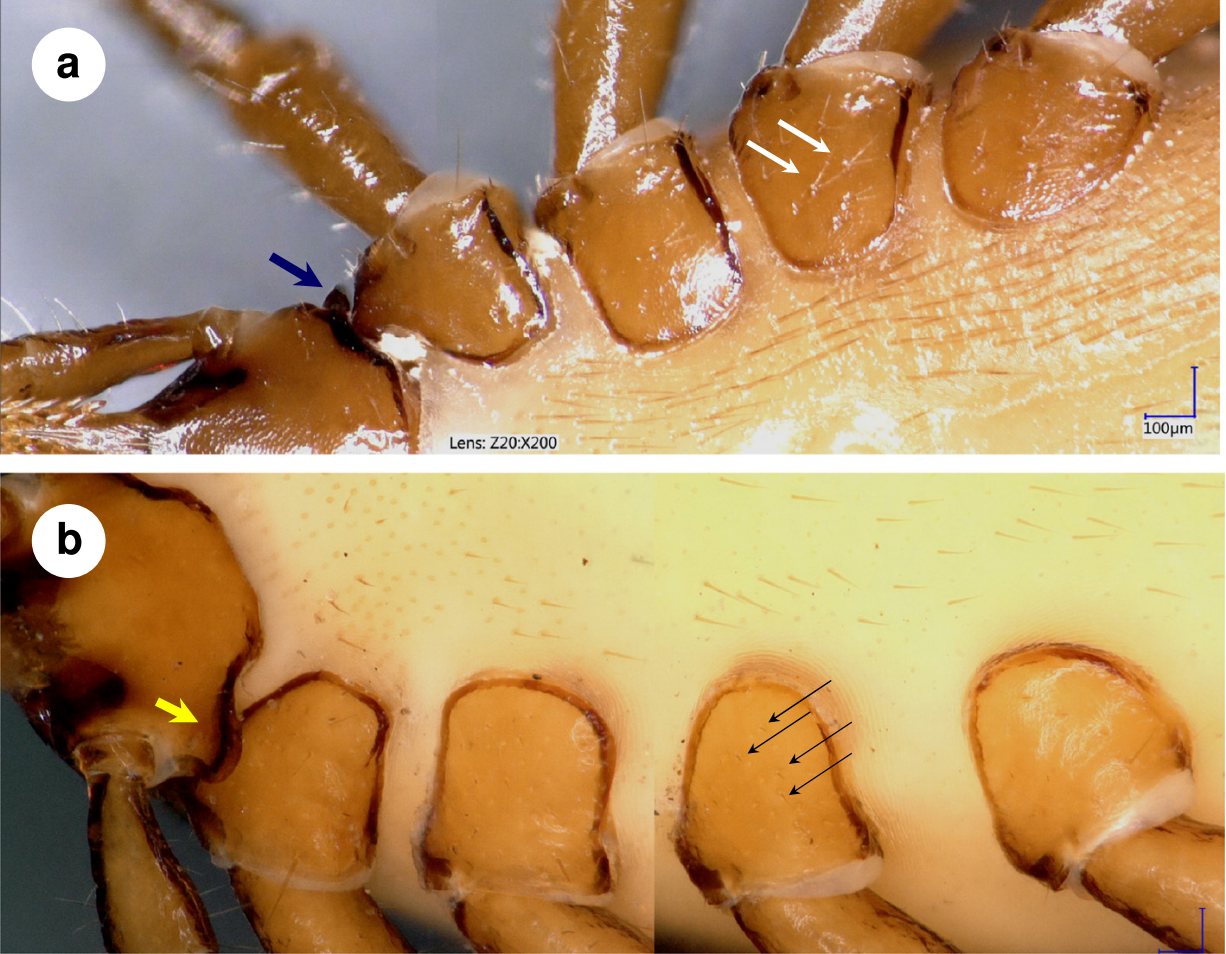
Ventral view of female **a** *Ixodes vespertilionis* and **b** *Ixodes collaris* n. sp. (holotype). **a** *I. vespertilionis* shows lateral flange on basis capituli (*blue arrow*) and a few, long coxal setae (especially on coxa III: *white arrows*). Note: V-shaped arrangement of some of these setae is due to reflection. **b** *I. collaris* n. sp. with ventral collar on basis capituli (*yellow arrow*) and multiple, short coxal setae (*black arrows*)

***Female*.** [Based on the holotype; Figs. 1a, 2a, c, d, 3b and 5/1.a-e.] Length of idiosoma (from half point between scapular apices to the posterior margin) 6 mm. Scutum elongate, reverse pentagonal in shape, broadest at mid-length (Fig. 1a), 2 mm long, 1.325 mm wide (shape index 1.5); scutum edge concave anteriolaterally, convex and almost rounded caudo-laterally, posteriorly (Fig. 1a). Cervical grooves moderately deep, approaching posterolateral scutal margin slightly posterior to level of maximum breadth. Punctuations scattered, more apparent anteriorly. Scutal setae situated only anteriolaterally and between scapulae, few, short (< 50 μm). Alloscutal setae dense, longer than scutal (*c.*100 μm or longer) next to anterior half of scutum and laterally on idosoma; posterior setae longest (>120 μm). Ventral idiosomal setae shorter (<100 μm) anteriorly to genital aperture and longer (>100 μm) posteriorly (Fig. 2a), encircling anus (Fig. 2c). Genital aperture between coxae III. Spiracular plates oval, with excentric opening (Fig. 2d).

Length of gnathosoma (from palpal apices to posterior margin of basis capituli) 1.08 mm, width of basis capituli dorsally 0.77 mm; ratio gnathosoma length to basis capituli width 1.4. Basis capituli dorsally triangular, posteriorly with *c.*50 μm thick transverse ridge continuing at sides dorso-ventrally (Fig. 1c). Longitudinal flanks laterally to porose areas (anteriorly and perpendicularly to transverse ridge), first diverging, then converging (maximum distance between flanks 0.56 mm). Porose areas longer than broad, anteriorly tapering, separated by broad (100 μm) interval (Fig. 1c). Semi-transparent and broad ridge (“collar”) present on both sides ventrally and caudolaterally on basis capituli, nearly horizontal in position, overlaying anterior part of coxae I (Fig. 3b). Palps slender, elongate (0.84 mm long): segments I, II, III dorsally measuring 80, 470 and 290 μm, respectively. Hypostome 0.55 mm long, 0.18 mm wide (ratio length to width 3), pointed, conical, bearing elongate denticles arranged as 3/3 to 4/4 (with decreasing size) towards apex.

Legs long, slender. Coxae lacking spurs (coxa I slightly produced caudomedially), with few long setae and numerous minute (< 20 μm) setae (Fig. 3b). Tarsus I 1.25 mm long. Haller’s organ open, elongate. Anterior pit sensillae (i.e. those closer to tarsal end), in a group, with a prominent sensillum.

**Fig. 4:**
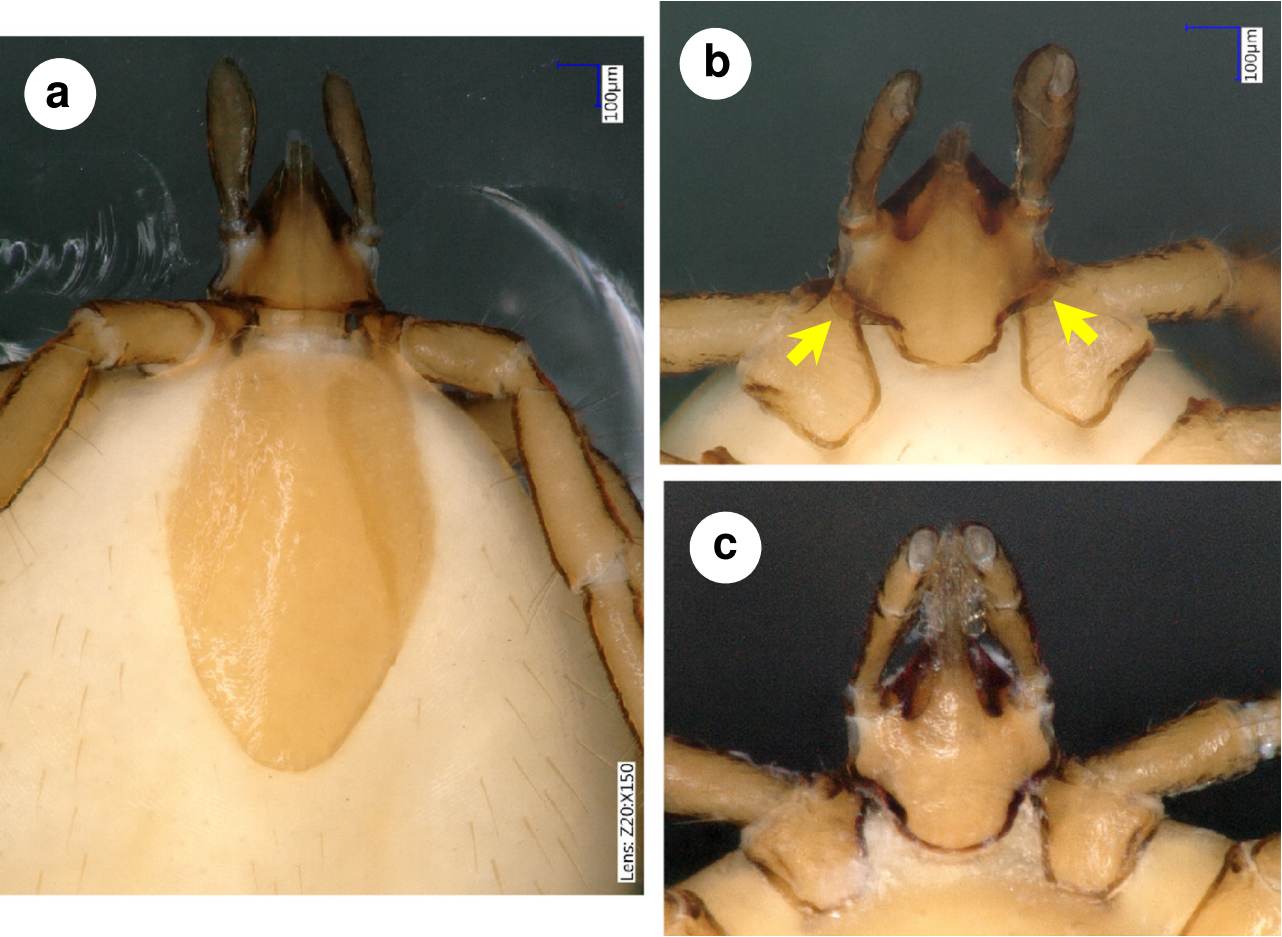
Nymphs of *Ixodes collaris* n. sp. (paratype No. 2) (**a**, **b**) and *I. vespertilionis* (**c**). **a** *I. collaris* n. sp., dorsal view. **b** *I. collaris* n. sp., gnathosoma, ventral view. Note semitransparent collars extending above the first coxae (*arrows*). **c** Gnathosoma of *I. vespertilionis*, ventral view

**Fig. 5:**
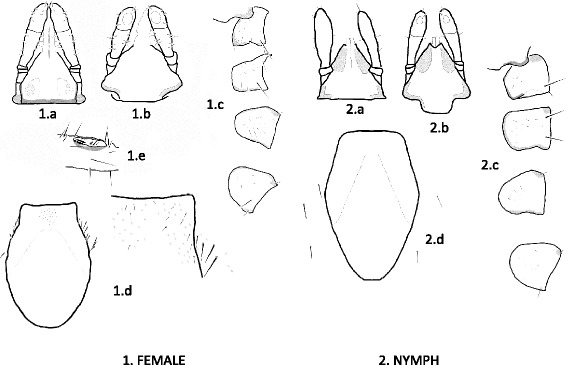
Drawings of structures with diagnostic importance in the female (1) and nymph (2) of *Ixodes collaris* n. sp. *Labels*: 1.a and 2.a, capitulum dorsal view; 1.b and 2b, capitulum ventral view; 1.c and 2.c, coxae (downward: I-IV) with the collar overlaying coxa I; 1.d and 2.d, scutum; 1.e, Haller’s organ

***Nymph*.** [Figs. 4a, b and 5/2.a-d.] Length of idoisoma 3.7 mm. Scutum elongate, reverse pentagonal (broadest at midlength), with slightly convex (almost straight) caudo-lateral edge (Fig. 4a). Transverse folds present on scutum anteriolaterally; punctuations scattered. Length of scutum 1.04 mm, breadth 0.67 mm, shape index 1.55. Cervical grooves moderately deep, approaching posterolateral scutal margin posterior to its maximum breadth. Alloscutal setae scattered, long, longest posteriorly (> 200 μm). Ventral idiosomal setae shorter (<100 μm) anteriorly and around anus. Spiracular plates oval.

Length of gnathosoma (from palpal apices to posterior margin of basis capituli) 0.65 mm, width of basis capituli dorsally 0.46 mm; ratio gnathosoma length to basis capituli width 1.4. Basis capituli dorsally triangular, posteriorly with transverse ridge continuing at sides dorso-ventrally. Caudolateral, semi-transparent, broad ridge (“collar”) present ventrally on both sides of basis capituli (Fig. 4b), overlaying anterior part of coxae I. Palps slender, elongate (0.45 mm): segments I, II, III measuring dorsally 50, 230 and 170 μm, respectively. Hypostome incomplete.

Legs moderately long, slender. Coxae lacking spurs (coxa I slightly produced caudomedially). Coxae with some long setae and few minute (< 20 μm) setae. Tarsus I 0.75 mm long.

### Differential diagnosis

Major morphological differences between females of *I. collaris* n. sp. and females of *I. vespertilionis* are the following. The scutum of *I. vespertilionis* (Fig. 1b) is posteriorly tapering, with a shape index exceeding 1.6 [4, 5] whereas the scutum of *I. collaris* n. sp. is posteriorly broad, with a shape index around 1.5 (Fig. 1a). Ventrally, the setae located anteriorly to the genital aperture are shorter than those located posteriorly in *I. collaris* n. sp. (Fig. 2a), whereas the setae of *I. vespertilionis* are similar in length both anteriorly and posteriorly to the genital aperture (Fig. 2b). The porose areas in *I. vespertilionis* are broader than long, separated by a narrow interval [4, 5], and surrounded by an anteriorly converging flank (Fig. 1d). However, the porose areas in *I. collaris* n. sp. are longer than broad, separated by a broad interval and surrounded by an initially divergent, then convergent flank (Fig. 1c). The female of *I. vespertilionis* has a dorsal transverse ridge posteriorly on the basis capituli that continues around the sides as vertical, outwardly directed flange (which is perpendicular to the hypostome) [4]. Adding to this, ventrally on the basis capituli of *I. collaris* n. sp. a broad, collar-like extension is present behind the palpal base caudolaterally (in a nearly horizontal/longitudinal direction). Furthermore, the coxae of *I. vespertilionis* female bear few long (100 μm or above) setae posteriorly [5] (Fig. 3a), whereas multipe short setae predominate on the coxae of *I. collaris* n. sp. (Fig. 3b). The arrangement of anterior pit sensillae in Haller’s organ is grouped in the case of *I. collaris* n. sp., but linear in the case of *I. vespertilionis* [6]. Regarding nymphs of *I. collaris* n. sp., the bilateral collars (Fig. 4b) clearly distinguish the new species from *I. vespertilionis* (Fig. 4c).

The female of *I. collaris* n. sp. also differs from the female of *I. ariadnae* in the shape of the scutum (anteriorly narrow *vs* anteriorly broad) and porose areas (longer than broad *vs* broader than long), the palpal shape (elongated *vs* broad), the presence (*vs* absence) of collar, the arrangement and length of coxal setae (predominance of short *vs* long setae) [6]. On the other hand, similarities between *I. collaris* n. sp. and *I. ariadnae* include the grouped (non-linear) arrangement of anterior pit sensillae in Haller’s organ, as well as the folded surface of nymphal scutum [6].

In contrast to the female of *I. collaris* n. sp., the female of *I. simplex* has anteriorly broad scutum, short and broad palps, and short legs with multiple long setae on coxae IV [6].

## Discussion

The genetic differences between ixodid bat ticks collected in Europe and Asia were shown to greatly exceed the limit of intraspecific sequence divergence [1]. In line with this, the morphological characteristics of *I. collaris* n. sp. described above, clearly support its distinct species status. Interestingly, while *I. collaris* n. sp. was apparently misidentified as *I. vespertilionis* in several parts of Southeast Asia, based on the analysis of both COI and 16S rDNA genes, this new species is phylogenetically closer to *I. ariadnae* than to *I. vespertilionis* [1].

Geographical separation of bat populations may be an important driver of the speciation of their ticks. In line with this, the geographical isolation of lesser horseshoe bats (*R. hipposideros*) north and south of the Pyrenees [7], was reported to entail high level of genetic difference between their ixodid ticks (*I. vespertilionis*) [1]. The main host of *I. vespertilionis* (*R. hipposideros*) and those of *I. collaris* n. sp. reported here (*R. affinis*, *H. pomona*) show allopatric distribution in Eurasia, i.e. the former occurs west of the Himalayas, whereas the latter two in and east of the Himalayas. This (and possibly other factors in niche segregation) may have acted as selective pressures towards divergent evolution of these bat species (which cluster separately in phylogenetic analyses: [1, 8]) and of their ixodid ticks, i.e. *I. vespertilionis* and *I. collaris* n. sp.

*Ixodes vespertilionis* has long been regarded as uncommon in south-east Asia [9], and this may explain why no detailed comparative morphological studies were carried out on this tick species in the region. The description of *I. vespertilionis* from Japan by Yamaguti et al. [10] indicates several shared features with *I. collaris* n. sp., e.g. the shape of the scutum, the presence of the ventral collar and the grouping of anterior pit sensillae in the Haller’s organ. Therefore, also taking into account the relatively close phylogenetic relationship of bat ticks from Vietnam and Japan [1] it is possible that *I. collaris* n. sp. also occurs in Japan. In addition, because *R. affinis* appears to be an important host of the newly described bat tick species (as shown here), and this bat species was reported to be the predominant host of *I. vespertilionis* in southern China [11], we hypothesise that *I. collaris* n. sp. has a broad geographical range in south-east Asia.

## Conclusion

In this study the female and the nymph of an ixodid bat tick species from Vietnam are described for the first time. The genetic and morphological differences between *I. vespertilionis* Koch, 1844 and these bat ticks from Vietnam justify the status of the latter as a distinct species *I. collaris* Hornok n. sp.

## Abbreviations

Not applicable.

## References

[1] Hornok S, Estrada-Peña A, Kontschán J, Plantard O, Kunz B, Mihalca AD, et al. High degree of mitochondrial gene heterogeneity in the bat tick species *Ixodes vespertilionis*, *I. ariadnae* and *I. sim*plex from Eurasia. Parasit Vectors. 2015;8:457. 10.1186/s13071-015-1056-2 PMC4573304 26382218

[2] Lv J, Wu S, Zhang Y, Chen Y, Feng C, Yuan X, et al. Assessment of four DNA fragments (COI, 16S rDNA, ITS2, 12S rDNA) for species identification of the Ixodida (Acari: Ixodida). Parasit Vectors. 2014;7:93. 10.1186/1756-3305-7-93 PMC3945964 24589289

[3] International Commission on Zoological Nomenclature (2012). Amendment of articles 8, 9, 10, 21 and 78 of the International Code of Zoological Nomenclature to expand and refine methods of publication. Zootaxa.

[4] Arthur DR (1956). The *Ixodes* ticks of Chiroptera (Ixodoidea, Ixodidae). J Parasitol.

[5] Feider Z (1965). Ixodoidea. Fauna of the Popular Republic of Romania.

[6] Hornok S, Kontschán J, Estrada-Peña A, Fernández de Mera IG, Tomanovic S, de la Fuente J (2015). Contributions to the morphology and phylogeny of the newly discovered bat tick species, *Ixodes ariadnae* in comparison with *I. vespertilionis* and *I. simplex*. Parasit Vectors.

[7] Dool SE, Puechmaille SJ, Dietz C, Juste J, Ibáñez C, Hulva P, et al. Teeling EC. Phylogeography and postglacial recolonization of Europe by *Rhinolophus hipposideros*: evidence from multiple genetic markers. Mol Ecol. 2013;22:4055–70. 10.1111/mec.12373 23889545

[8] Li G, Liang B, Wang Y, Zhao H, Helgen KM, Lin L, Jones G, Zhang S (2007). Echolocation calls, diet, and phylogenetic relationships of Stoliczka’s trident bat, *Aselliscus stoliczkanus* (Hipposideridae). J Mammal.

[9] Petney TN, Keirans JE (1994). Ticks of the genus *Ixodes* in South-east Asia. Trop Biomed.

[10] Yamaguti N, Tipton VJ, Keegan HL, Toshioka S (1971). Ticks in Japan, Korea and Ryukyu Islands. Brigham Young Univ Sci Bull Biol Ser.

[11] Bush SE, Robbins RG (2012). New host and locality records for *Ixodes simplex* Neumann and *Ixodes vespertilionis* Koch (Acari: Ixodidae) from bats (Chiroptera: Hipposideridae, Rhinolophidae and Vespertilionidae) in southern China. Int J Acarol.

